# Notes and News

**Published:** 1890-09-13

**Authors:** 


### Notes and News.

Collected and Communicated.

Hospital Saturday has made an excellent start in Brighton. Glorious weather doubtless contributed greatly to the success of the venture, but nothing was left undone on the part of its promoters which could in any way be likely to swell the amount collected. Processions, entertainments, bands, and decorations were all used freely to advertise the cause of charity, and the result was the handsome sum of nearly £1,200. Upon so successful a termination to their first Hospital Saturday, the Brighton people are heartily to be congratulated, and it may surely be one of the most pleasant recollections of Alderman Manwaring's mayoralty to have inaugurated such a noble institution with such brilliant results.

The Quarterly Court of the Belfast Royal Hospital was held on August 25th. The medical report showed that, as in previous quarters, the resources of the hospital in the treatment of patents both intern and extern had been taxed to the utmost limit, the number of cases altogether reaching the total of 5,924. A slight improvement was to be observed in the finances, and it had been possible to effect a reduction in the indebtedness of the institution; but a great deal will yet require to be done before the hospital funds can be said to be placed on a sound footing. A somewhat melancholy interest was attached to the proceedings from the fact that since the last meeting the committee had to record the deaths of Dr. D. B. Croker and Dr. James Smith. It is peculiarly distressing to hear of the large number of applications for the admission of consumptive patients to the Throne Hospital which were compelled to be refused in consequence of the want of accommodation. It is deeply to be regretted that the generous offer made by a citizen to enlarge the Throne Hospital could not be taken advantage of, the conditions attaching not having been complied with; but it is to be hoped the remarks made at the meeting will have the effect of stirring up the minds of the public to the urgent necessity of making much fuller provision than that now existing for the care of those who become victims to the terrible malady of consumption. We hope the eloquent and earnest appeal of Archdeacon Seaver and other gentlemen will reach the hearts of those who can be moved by suffering.

As a memorial to the late Alderman Brooksbank, who for thirty-seven years was a member of the weekly board of the Sheffield Infirmary, and its president for the last seventeen years of that period, it has been decided to start a fund to purchase mechanical appliances and instruments for patients requiring such assistance. Such a fund must necessarily be of the greatest advantage to patients who have received treatment at the infirmary, and a more practical memorial, or one more likely to have been approved by Mr. Brooksbank himself, could hardly have been suggested.

The annual meeting of supporters of Stockton Surgical Hospital took place on August 26th, Mr. G. M. Watson, J.P., in the chair. During the year the number of in-patients treated was 557, or an increase on the previous year of 84. The in-patients were under treatment 26½ days each, and the cost of their maintenance was 2s. 3½d. per head per day, against 1s. 10¼d. per head per day last year. The death-rate among the in-patients was 2·10 per 100; but five patients died within a few hours after admission from the effects of serious accidents. The increase in the number of in-patients treated was doubtless due in a great measure to the greater number of hands employed at the various works, and the beds had consequently been frequently all occupied. The number of out-patients treated during the year was 1,597. The new wing was rapidly approaching completion, and in the course of a few weeks would be ready for occupation. The hospital has a balance of £2,950 at its bankers, but this will be mostly absorbed by the new wing.

Sheffield General Infirmary has a satisfactory report of its ninety-third year's work, but £1,500 is a serious discrepancy to show between the income and expenditure, and where, as in this case, the amount is on the wrong side, it must be a matter of grave regret. The deficiency is mainly accounted for by alterations in the sanitary arrangements and the renewal of 105 worn-out bedsteads. Despite the heavy outlay required, it has been decided to complete the improvements which have been begun. During the year the in-patients numbered 1,687, out-patients 3,301, accidents and emergencies 11,810, and dental cases 2,237, a total number of 19,035 cases of all kinds. There is a decrease in the number of in-patients, mainly owing to the fact of several wards having been closed for a considerable time for alterations. The available beds now number 200, of which 44 are devoted to children's cases. The "Overend Convalescent Fund" enabled 151 patients to be sent from the infirmary for change and rest.

The West of Scotland Seaside Convalescent Homes at Dunoon celebrated their coming of age on August 28th last. The founder, Miss Beatrice Clugston, who also originated the Lenzie Homes, and the Broomhill Home for Incurables at Kirkintilloch, has not lived to see the institution for which she worked so well attain its majority, but it has proved a fitting monument to her life of noble self-devotion. Convalescent homes need no eulogy, the value of their work is too well recognized for that. The Dunoon institution has now accommodation for 200 inmates, and its income last year was £5,758. During the 21 years of its existence, the contributions to it have been £102,574, and the expenditure £97,370, thus leaving a balance in hand of £5,200. The average cost per patient for the last five years has been 5s. 3¾d. per head per week, or 9¼d. per day. In all, some 45,000 patients have been treated, of whom only about 2,000 were discharged not improved, and the deaths during the whole period have been 55, or one in 820. After the general meeting, which was presided over by Sir James King, the directors and guests were entertained at dinner.

The annual statement of Richmond Union lies before us—a statement which tells of careful and economical expenditure, and which supplies full details of work done. The most interesting column is that headed "Cause of Relief." The most frequent reason given under this heading is old age, the second illness; then comes loss of work, widow, labour test, deserted by husband, husband in prison, husband in hospital, blind, and finally funeral. Such are the sorrows of the poor which drive them to seek the carefully calculated doles of the guardians. The cost of a pauper funeral is not great; where a child, name unknown, is found dead the funeral costs 13s. 9d.; whereas the funeral of a man seems to cost £1. How many tragic tales lie hid in these crisp, concise statements of "Cause of Relief"!

---

The Herbert Convalescent Home, at Bournemouth, was open 46 weeks as against 36 weeks last year, and the total number of convalescents admitted was 376 (men 177 and women 199) as against 270. This is the highest number yet reached in any one year. The average stay of convalescents in the home was 27½ days, and the average cost of each inmate, convalescents and household, for provisions, necessaries, and so forth, was 10s. 6d. per week, as against 11s. 1d. last year, while the gross cost of maintaining each bed, inclusive of salaries and all ordinary expenses, was 16s. 6d. per week, as against 18s. 11d. last year. It is satisfactory to note that the subscriptions show an increase of £60, and the donations and collections, which last year showed an increase of £160, have again increased by nearly £100. Generosity, aided by good management, have enabled the accounts to be closed with a small balance in favour of the home.

---

The Committee of Management of the Salisbury Infirmary have presented their report for the 124th year since the foundation of the institution. The report shows that the in-patients numbered 855, as against 812, and the out-patients 1,640, as against 1,743 last year, and there were 38 deaths. Twenty-seven patients were sent from the infirmary to the Herbert Convalescent Home at Bournemouth, as against 12 the previous year. Attention was drawn to the inferior accommodation of the nursing staff in respect of the absence of a day-room, and the total want of privacy in the dormitories, and it has also been arranged that in addition to nursing students, who are admitted for twelve months on payment of a fee of five guineas, probationers are to be appointed, who are willing to pay ten guineas, and engage for two years instead of one, thus securing to the infirmary the benefit of the experience they acquire and the training they receive during the first half of that period.

---

Dr. Newsholme says that a vast incubus of ignorance and indifference regarding sanitary matters has still to be removed, and that the main hope for the future lies in the education of the children. They must be taught the great essentials of healthy existence. Certain it is that little will be done till the people themselves stand out for healthy dwellings, and until they realise how much disease is preventable this cannot be. The Sanitary Congress passed a resolution that the study of hygiene ought to be pursued in schools, and so it undoubtedly ought. We hear a great deal about technical schools and classes for the working classes; let the promoters of these excellent institutions listen to the advice of the congress, and let them include amongst their other instruction some lessons on simple hygiene, and teach their pupils that the sunlight and the fresh air were not sent us for nothing. Are we not all agreed about the necessity for moral elevation of the people? Surely nothing will help this sooner than the improvement of the conditions in which they live!

The British Ophthalmic Hospital, Jerusalem, is a growing institution, and its sixth report is very pleasant reading. Originally a dispensary for out-patients only, the hospital or in-patient department has steadily increased, and it is hoped that very shortly the beds will number 20 at least. Last year the in-patients admitted numbered 240, while the total attendance of out-patients was 7,383, of whom 2,833 were new cases. Operations numbered 324, of which 266 were principal and 58 minor, and that the results of the treatment to be obtained at the institution are satisfactory is amply proved by the increase in the applications for admission. The hospital is a department of the Grand Priory of the Order of St. John of Jerusalem in England, upon which the Royal Charter of Incorporation has been conferred since the issue of the previous report, and of which Her Majesty the Queen has become the Sovereign Head and Patron.

---

An American paper pays the tribute of a tear to the departed race of good old-fashioned country doctors. It maintains that though there are plenty of clever, active, perhaps even disinterested country medical men now, they are for the most part physicians rather than doctors; and it is more than a mere whim to regard the two words as by no means synonymous. The old type has given place to a set of men more alive to their own interests, more imbued with the restless spirit of the age, more accustomed to stand upon purely business rather than friendly relations with their patients. The older men had many theories both medical and religious at which the younger men smile contemptuously; they gave drugs with a generous profusion which it is now appalling to recall; they, perhaps, comforted their sick with dogmas as much out of date now as their medical methods; but their strong, helpful spirit, their manly unselfishness, their patient sympathy, were beautiful and noble things, by the loss of which the world is infinitely poorer. It is useless to wish them back, since the march of progress has made their existence well-nigh impossible; but who can look back to their day and not regret these unregarded heroes?

